# Polyethylene Terephthalate (PET) Recycled by Catalytic Glycolysis: A Bridge toward Circular Economy Principles

**DOI:** 10.3390/ma17122991

**Published:** 2024-06-18

**Authors:** Andra-Cristina Enache, Ionela Grecu, Petrisor Samoila

**Affiliations:** “Petru Poni” Institute of Macromolecular Chemistry, 41A Grigore Ghica Voda Alley, 700487 Iasi, Romania; humelnicu.andra@icmpp.ro (A.-C.E.); grecu.ionela@icmpp.ro (I.G.)

**Keywords:** plastics, packaging, R-imperatives, chemical recycling, monomer yield, heterogeneous catalysts

## Abstract

Plastic pollution has escalated into a critical global issue, with production soaring from 2 million metric tons in 1950 to 400.3 million metric tons in 2022. The packaging industry alone accounts for nearly 44% of this production, predominantly utilizing polyethylene terephthalate (PET). Alarmingly, over 90% of the approximately 1 million PET bottles sold every minute end up in landfills or oceans, where they can persist for centuries. This highlights the urgent need for sustainable management and recycling solutions to mitigate the environmental impact of PET waste. To better understand PET’s behavior and promote its management within a circular economy, we examined its chemical and physical properties, current strategies in the circular economy, and the most effective recycling methods available today. Advancing PET management within a circular economy framework by closing industrial loops has demonstrated benefits such as reduced landfill waste, minimized energy consumption, and conserved raw resources. To this end, we identified and examined various strategies based on R-imperatives (ranging from 3R to 10R), focusing on the latest approaches aimed at significantly reducing PET waste by 2040. Additionally, a comparison of PET recycling methods (including primary, secondary, tertiary, and quaternary recycling, along with the concepts of “zero-order” and biological recycling techniques) was envisaged. Particular attention was paid to the heterogeneous catalytic glycolysis, which stands out for its rapid reaction time (20–60 min), high monomer yields (>90%), ease of catalyst recovery and reuse, lower costs, and enhanced durability. Accordingly, the use of highly efficient oxide-based catalysts for PET glycolytic degradation is underscored as a promising solution for large-scale industrial applications.

## 1. General Introduction

### 1.1. Background: Plastic Pollution Scenario

**Figure 1:**
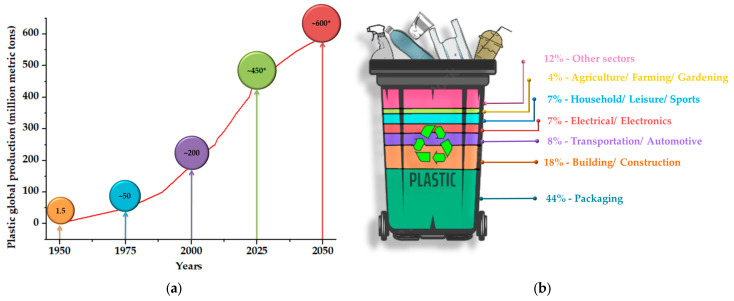
(**a**) Worldwide production of plastics from 1950 to 2022, with projections (*) for the period 2025–2050 (in million metric tons) [3,4,5]. (**b**) Distribution of the global plastics use in 2021 by sector of application (numeric data from [9]).

Plastic pollution is one of the most serious issues confronting the modern world, and it has been compounded in recent years by the COVID-19 pandemic, which has resulted in the overuse of personal protective equipment (e.g., masks, gloves, aprons, face shields, and disinfection bottles) [1,2]. Global plastic production has continuously increased over the past 70 years, from 2 million tons in 1950 to 400.3 million metric tons in 2022, as illustrated in Figure 1a [3,4]. The most concerning data indicate that over half of the world’s plastic manufacturing has been commercialized in the last 20 years, and it is predicted to expand to almost 600 million metric tons in 2050 (Figure 1a) [5]. These data are mainly the consequence of the rapid advancement of technology in response to shifting material demands of the world’s growing population [6].

Plastic materials are widely used in a variety of industries, such as consumer goods, electronics, transportation, packaging, medical equipment, construction, and others [7,8]. As can be observed in Figure 1b, “packaging” is the largest sector of usage for plastics, accounting for nearly 44% of annual global production in 2021 [9]. Hence, the increased use of plastic, mainly for food and beverage packaging, is due to advantages such as low production costs and extended shelf life, as well as plastic’s unique properties (strength, durability, light weight, electrical and thermal insulation, chemical stability, and corrosion resistance) [6,10,11].

Despite the benefits they provide, most of the monomers used in the manufacture of plastic materials (e.g., ethylene and propylene) are derived from fossil hydrocarbons and are not biodegradable [8]. Consequently, instead of degrading, plastic debris accumulates in landfills or in the natural environment, causing serious problems to living organisms [6,12]. It was estimated that 12 million tons of plastic waste is dumped into the ocean annually, which is roughly the same as one dumpster truck every minute [13]. The most well-known example is the Great Pacific Garbage Patch, which is thought to contain 80,000 tons of plastic and 1.8 trillion plastic particles floating in the open ocean [14]. Because the marine and the terrestrial environment are inextricably linked, alterations to one system can have a detrimental impact on the other [15]. Furthermore, the extraction and transportation processes of the crude oil contribute to the emission of greenhouse gases. Moreover, the purification and fabrication of plastic materials, along with the disposal of plastic waste, are procedures that can also impact climate changes [16,17]. Thus, as a result of inadequate waste management and recycling methods, plastic pollution is considered the “One health issue of global scale” [18], generating ecological repercussions that threaten both human and animal health [7,18].

### 1.2. Research Motivation: The Widespread Use of PET as a Packaging Material

**Figure 2:**
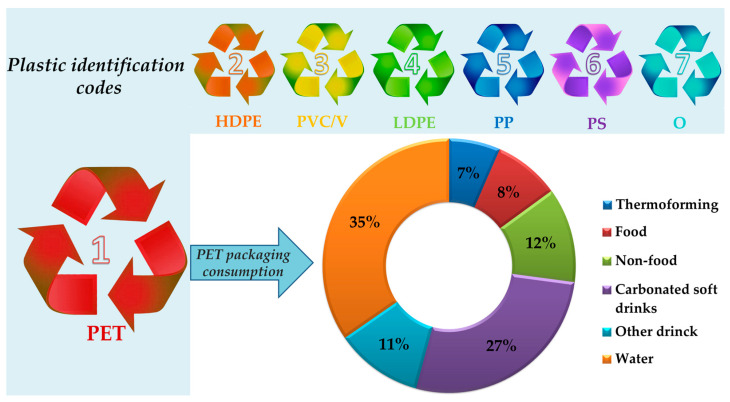
The identification codes (recycling symbols) of the main thermoplastics: PET—poly(ethylene terephthalate); HDPE—high-density polyethylene; PVC—poly(vinyl chloride); LDPE—low-density polyethylene; PP—polypropylene; PS—polystyrene; and O—other plastics; worldwide PET packaging consumption in 2019 by categories (numeric data from [23]).

Although a huge range of plastics are known to be used in packaging, the most used materials are the thermoplastics. These polymers are based on relatively weak intermolecular interactions and may soften in the presence of heat and harden in the absence of it—a property that is particularly crucial for effective recycling [11]. Polyethylene terephthalate (PET), high-density polyethylene (HDPE), poly(vinyl chloride) (PVC), low-density polyethylene (LDPE), polypropylene (PP), and polystyrene (PS and EPS) are the main six thermoplastics dominating the market (>80%) [13,19]. Their identification symbols are evidenced in Figure 2. Among other thermoplastics, PET is almost exclusively used in packaging, mainly for beverages (about 73% worldwide in 2019, as graphically depicted in Figure 2).

In Europe, single-use beverage bottles represent the majority of PET usage, made by transparent (78%), colored transparent (20%), and opaque (2%) PET. Unlike the small amount, the latter interferes with current PET recycling strategies, raising considerable challenges in recycling and preservation of its properties [13]. This is mainly because of its composition, which includes TiO_2_ particles, various PET grades, and additional impurities (such PE and inorganic materials). As a result, researchers focused most on methods for upcycling the opaque PET waste, such as a five-stage acetolysis process (mechanical shredding, acetolysis, hydrolysis, repolymerization, and extrusion), which included a decolorization step for colored PET [20], integration into recycled polypropylene using a microfibrillation technique [21], or designing new materials with improved rheological and mechanical properties by extrusion and treatment with Joncryl [22]. Given the predominance of transparent PET in single-use beverage bottles and the variety of available recycling methods for PET waste, this review primarily focuses on transparent PET.

**Figure 3:**
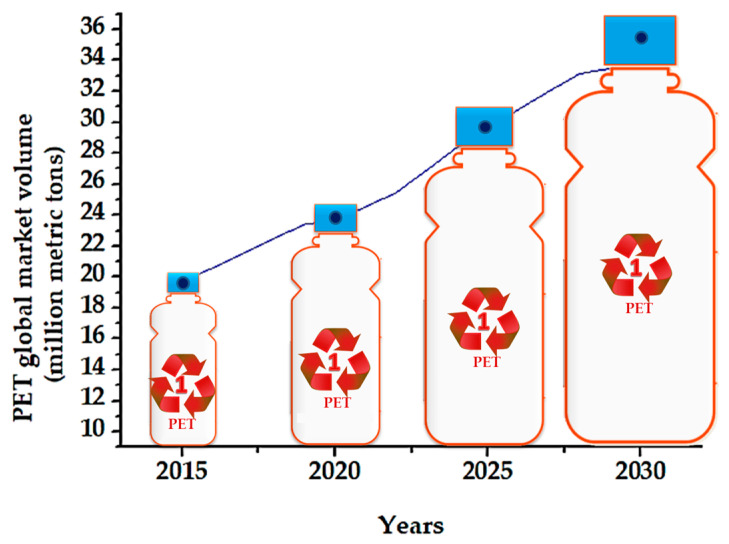
Worldwide market volume of PET (in million metric tons) from 2015 with predictions till 2030 (numeric data from [26]).

Hence, PET’s popularity as a packaging material stems from several properties, such as transparency, food safety, cleanliness, impact strength, UV resistance, durability, cost-effectiveness, and barrier properties [13,24,25]. However, the increase in the world market volume over recent years (from 19 million metric tons in 2015 to approximately 25 million metric tons in 2022), as detailed in Figure 3, has had a detrimental effect on the environment. Most concerning is that the PET volume is expected to reach 36 million metric tons in 2030 [26]. As a result, during the processes of production, application, and disposal, significant amounts of PET have been released into the environment, and the accumulation of PET wastes is steadily rising, thus endangering ecosystems all over the world [27]. For example, it has been predicted that, out of every million PET plastic bottles sold globally every minute, more than 90% ultimately end up in landfills or the ocean, taking hundreds of years for PET to fully decompose in the environment [17,28]. In 2020, 7297.7 kilotons of PET were consumed worldwide, with just 23% recycled, 35% incinerated, and 44% landfilled or disposed into the environment, according to a study conducted across 12 global regions (41 countries that, together, manufacture over 95% of the world’s PET) [29]. Also, through partial decomposition, PET-derived microplastics indeed become prevalent in aquatic and marine ecosystems. Subsequently, these micropollutants, as bigger fragments and microplastics, are consumed by living organisms, inducing health issues [17].

### 1.3. The Challenging Framework of PET Recycling

The ongoing challenge lies in discovering ways for the efficient prevention, collection, and management of plastic packaging waste (specifically PET). Thus, the adoption of zero-waste circular economy (CE) approach within the plastic packaging sector is recognized as a crucial stride toward its tangible realization [11,13,30]. In accordance, the acceleration of plastic circularity has attracted a lot of interest from governments, policymakers, and intergovernmental organizations, as well in the research literature of the last years [11,31,32,33,34,35,36,37,38,39,40]. Furthermore, it should be mentioned that, in addition to recycling solutions, improving consumer behavior is the key to promoting PET circularity. In this context, Walzberg et al. explored in-depth behavioral interventions targeting PET bottle recycling, demonstrating how simplifying recycling operations and educating consumers can improve the number of recyclables collected and significantly lower contamination levels [41].

**Figure 4:**
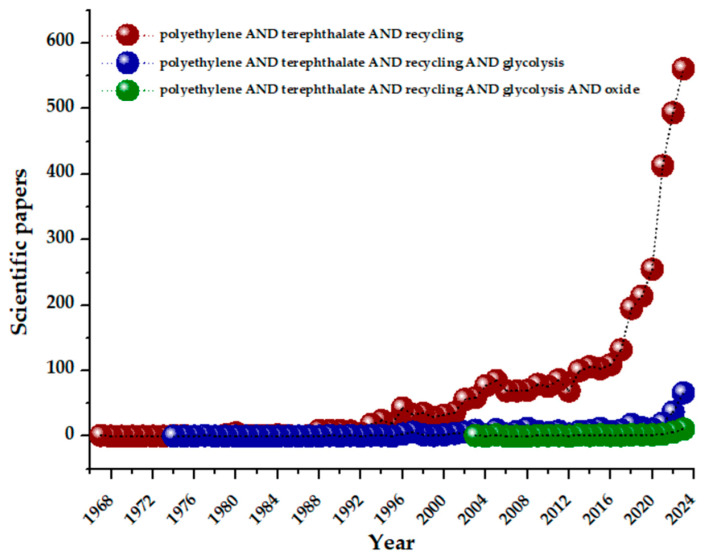
Scientific papers published from 1967 to 2023 (Scopus search on 23 January 2024) containing “polyethylene terephthalate”, “recycling”, “glycolysis”, and/or “oxides” in title, abstract, or keywords.

PET became lately the most globally recycled plastic, being recognized for its near-infinite recyclability [24,42]. The idea of recycling PET polymer has been researched since 1967. During this time, it was demonstrated that changes induced in PET by heating can be almost totally reversible [43]. As can be observed in Figure 4, research focused on PET recycling scarcely increased from 1967 to 1992. Following that, there was a period of modest expansion (1990–2016), culminating in the last 6 years with an accelerated increase (of six times more) of scientific works approaching the PET recycling concept. This is due to the pressing requirement for reducing the worldwide pollution caused by plastics, especially PET. Thus, different methods and strategies were proposed over the years (e.g., primary, secondary, tertiary, and quaternary methods), which are discussed in brief in this review, along with the concept known as “zero-order” recycling (direct reuse of PET waste) [44].

Based on recent studies (Scopus database), glycolysis is seen as an effective method for PET chemical recycling because it operates under milder conditions, uses less-volatile solvents, and yields a relatively pure monomer [45,46,47,48]. Consequently, by employing ethylene glycol (EG) as a solvent, one can add value to waste by transforming PET into the commonly utilized monomer, bis(2-hydroxyethyl) terephthalate (BHET) [44,47]. The depolymerization of PET by glycolysis was first approached in 1989 (Figure 4). Notably, the optimal results for recycled post-consumer soft-drink bottles were obtained by using excess ethylene glycol at 190 °C in the presence of a zinc acetate catalyst [49]. Furthermore, Figure 4 shows current research using oxide-based catalysts in PET glycolysis as a novel topic. It is noteworthy that, in recent years, metal oxide catalysts emerged as economically viable options due to their robust mechanical strength and ease of preparation and separation, making them highly suitable for large-scale applications [50]. For instance, a research study from 2003 highlighted the use of dibutyltin oxide (DBTO) as a catalyst in PET waste glycolysis, resulting in hydroxyl telechelic PET oligomers [51]. These oligomers hold potential for further manufacturing polyester-ethers with thermoplastic elastomer properties. A subsequent study further supported these findings a couple of years later [52].

Given the recent increase in interest and relatively limited studies in this area, a comprehensive review is essential to highlight the role of oxide-based catalysts in PET glycolysis, particularly in promoting the material circularity.

### 1.4. Novelty and Goal of the Review

To the best of our knowledge, this is the first review to provide comprehensive coverage of the fundamental aspects of effective PET waste management by bringing together (i) essential insights into the chemistry and physical properties of PET; (ii) trending strategies for PET management in a circular economy framework; (iii) an up-to-date evaluation of the current existing PET recycling methods, based on mechanical or chemical processes; (iv) an in-depth investigation of glycolysis, specifically heterogeneous catalyzed glycolysis, as most advantageous chemical method in obtaining high-yield depolymerization for the recovery of pure monomer; and (v) a condensed analysis of the most recent literature regarding the potential of oxide-based catalysts in the efficient recycling of PET on a large scale. Overall, it represents a unique and thorough exploration aligned with circular economy principles, making it a valuable contribution to the field.

In a practical way, the attractive performance in terms of depolymerization rate and monomer selectivity of novel oxide-based catalysts in glycolysis of PET are highlighted, being essential for producing high-quality recycled raw materials. To strengthen its unique approach, this review delves into the dual function of these oxide-based catalysts, both as catalysts and as supports for other catalysts, offering valuable perspectives on their potential for large-scale industrial applications.

To ensure comprehensiveness in the review, a thorough search across multiple databases (Scopus, ScienceDirect, PubMed, Web of Science, and Google Scholar) was conducted to gather a broad spectrum of literature, which aligns with the multidimensional nature of current research.

## 2. PET Chemistry and Physical Properties

**Figure 5:**
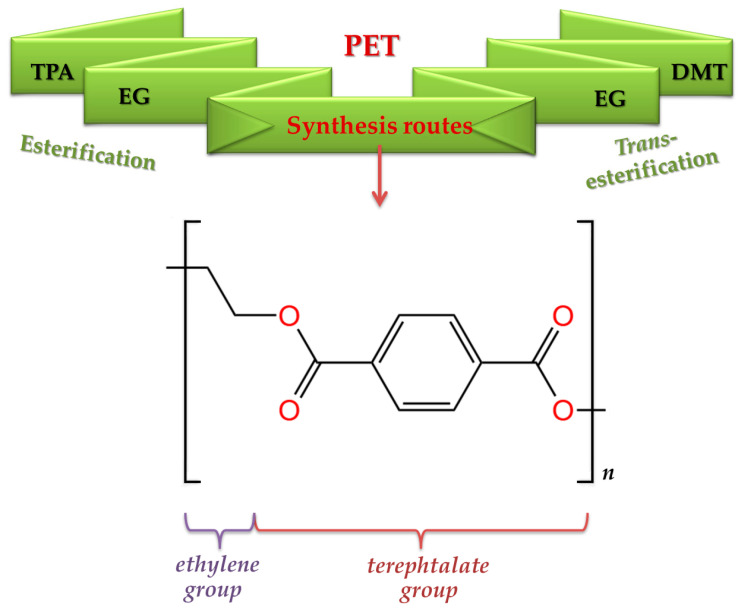
Synthesis routes for PET by esterification and *trans*-esterification and representation of repeating structural unit in the polymer chain (according to [54,55]).

Polyethylene terephthalate (PET) or 2-methoxyethyl-4-acetyl benzoate (IUPAC nomenclature) is a thermoplastic polyester with chemical formula and repeating unit structure detailed in Figure 5. PET, commonly employed for bottles, generally comprises approximately 100–140 repeating units [53]. However, the elemental composition of PET generally includes approximately 60% carbon, 30% oxygen, and 4% hydrogen by weight, with negligible ash content [17]. As indicated in Figure 5, the synthesis of PET typically involves two main routes: direct esterification (one-step process) of terephthalic acid (TPA) and ethylene glycol (EG); or *trans*-esterification of EG and dimethyl terephthalate (DMT), followed by the polycondensation of the obtained bis-hydroxyethyl-terephthalate (BHET) monomer [54,55].

Thus, a linear polymer containing both terephthalate and ethylene groups was finally obtained (Figure 5). PET’s aromatic ring is responsible for its strength, while the ethylene group provides flexibility. It should be mentioned though that the reversibility of the esterification reaction is considered the utmost importance for understanding PET’s behavior [53].

**Figure 6:**
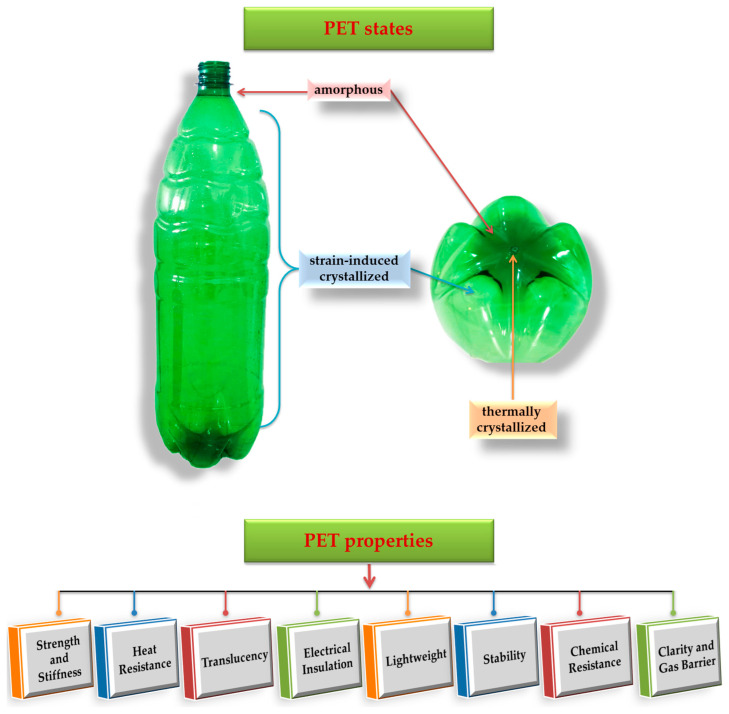
Distinct states of PET present in a stress-blow molded bottle (adaptation after [53]) and main PET properties (according to [55,56,57]).

According to McKeen et al. (2010), PET can be synthesized in two forms, namely amorphous and semi-crystalline [56]. Amorphous PET is characterized by a lack of a regular, ordered structure in its molecular arrangement, resulting in transparency. By contrast, semi-crystalline PET exhibits a partially ordered molecular structure arrangement, leading to opacity and a white appearance. However, Brandau et al. (2012) describe three distinct forms for PET, considering the division of the crystallized form of PET based on the crystallization method. Apart from the amorphous state usually found in preforms or molten plastic resin, PET can be manufactured in two additional states: thermally crystallized and strain-induced crystallized [53]. In the thermal crystallization process, crystals are initiated from a focal point, known as the nucleation site (e.g., resin pellets), and expand in a spherically organized pattern, radiating outward. Conversely, when strain-induced crystallization occurs (e.g., during stretch-blow molding), PET chains align in the direction of tension, creating an ordered linear structure across the stressed area [53]. This alignment is commonly observed in PET bottle walls, as illustrated in Figure 6.

Furthermore, as illustrated in Figure 6, PET material exhibits commendable properties. By comparison with other thermoplastics used in the packaging sector (e.g., HDPE), PET exhibits higher glass transition and melting temperatures. In addition, the exceptional clarity and translucency of PET is comparable to that of glass, but it brings an advantage in terms of safety. PET has also proven to be an effective barrier against oxygen, carbon dioxide, various odors and flavor compounds, and hydrocarbons [57]. Other advantages of PET include exceptional strength, stiffness, electrical insulating characteristics, resistance to various chemicals and water, stability due to minimal water absorption, and lightweight (economic transportation) [55]. These properties contribute to its widespread popularity, making PET one of the most widely used thermoplastics in the packaging sector. Furthermore, PET stands out as a recyclable choice that provides a superior performance compared to alternative packaging materials (e.g., glass bottles, metal cans, paperboard cartons, and various other plastics) [25]. Thus, within the framework of the circular economy (CE), understanding the behavior of PET as a material is essential in order to promote and advance the CE principles.

## 3. PET Management in a Circular Economy Framework

Since 2013, the Ellen MacArthur Foundation (EMAF) has offered a comprehensive perspective on the circular economy (CE). This involves linking essential concepts from diverse viewpoints, emphasizing that the CE aims to be an industrial economy centered on restoration, by both intention and design [58]. In other words, as opposed to the linear economy (take-make-dispose), CE advocates for the closure of loops in industrial systems with the goal of reducing waste to landfill, the energy input and raw resources [59,60]. Different R-imperatives have described practical strategies over the years, beginning with the 3R strategy (reduce, reuse, and recycle), which is asserted to align with the principles of the EMAF [31]. Detailed insights into these R strategies are provided by some reviews [31,61], showcasing variations in the number of Rs (ranging from 3R to 10R) and in terminology (with Reike et al. (2018) identifying 38 different “re-” words [61]). However, in the context of addressing zero plastic pollution in the CE context, various R-scenarios were envisioned to project the reduction of PET waste by the year 2040, as summarized in Table 1.

### 3.1. The 3R Framework

In order to shift away from a linear model of PET consumption and disposal to a circular approach that maximizes resource efficiency and minimizes waste, Geueke et al. (2018) considered the three significant waste management strategies, namely reduction, reuse, and recycling (3R strategy), as given in Table 1 [59]. Reduction involves the curtailment of raw material demand, energy consumption, material-use (e.g., single-use water bottle), and waste production, making it a “green” and impact-free approach. Implementing regulatory measures, such as mandatory charges on free plastic bag distribution, can contribute to reducing PET waste. Additionally, resource reduction often leads to cost savings [66]. Reusing products or components is similarly considered environmentally friendly (e.g., returning PET bottles by consumers) [60,76]. The recycling option, specifically the use of recycled material in lieu of virgin material, is widely recognized as a beneficial option. This approach is not only instrumental in conserving energy, resources, and emissions but also serves to diminish the environmental repercussions associated with material consumerism [59].

### 3.2. The 4R Framework

To mitigate the adverse impacts of PET pollution, Lau et al. [77] and Damayanti et al. [78] highlighted four adjusted strategic approaches, namely reduction, substitution, recycling, and disposal. In this regard, implementing novel interventions such as exploring alternative materials to replace conventional PET (substitution), increasing the collecting capacity through improved waste management, and adopting measures to curtail post-collection environmental leakage (dispose) is additionally proposed [77,78].

**Figure 7:**
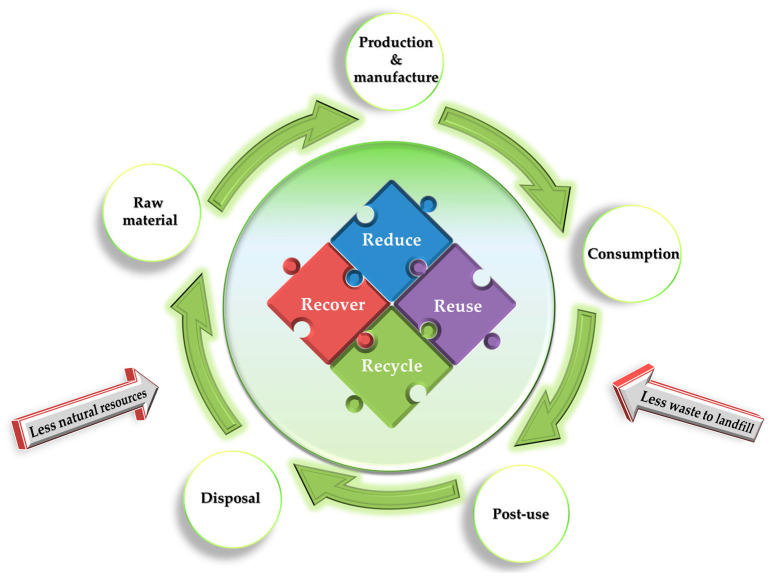
Schematic representation of 4R strategy for PET management in a circular economy framework.

However, as indicated in Table 1, alongside the 3R framework, the recovery of resources is also considered a strategically key component [63]. Accordingly, a circular economic model based on the 4R (reducing, reusing, recycling, and recovering) [64,65,66] was considered for an effective PET management. Figure 7 presents a schematic depiction of the life cycle of PET within the context of a 4R circular economy framework. This schematic representation demonstrates a holistic approach to PET management in a circular economy, emphasizing the interconnected nature of these strategies (4R strategy) to the cyclic processes involving PET production, manufacturing, consumption, post-use, disposal, and the recovery of raw materials. It is worth observing that the consumption of PET products underscores the significance of both minimizing single-use plastic and addressing post-use considerations, such as reuse or repair, before eventual disposal as waste. In addition, the recovery of raw materials (e.g., PET monomers) by methods like chemical recycling and bringing them back into the PET production cycle is essential for a close-loop system, contributing to a circular economy.

### 3.3. The 5R Framework

Yoshioka et al. (2015) mentioned, however, the expansion of the 3R strategy to a 5R framework by introducing the redesign and recovery of energy [62]. For instance, redesigning PET products to simplify concepts and avoid complex mixed materials ensures easy separation during the recycling process. According to Nandi et al. (2023), an effective approach for achieving circularity with PET waste involves adopting the 5R framework, which encompasses refusing, reducing, reusing, recycling, and rot (incorporating organic decomposition) [67]. The principle of refusing entails a conscious effort by individuals and businesses to avoid using environmentally unfriendly PET products, thereby mitigating waste generation. In addition, exploring biodegradable or compostable alternatives to PET and promoting practices that facilitate the decomposition of organic waste is encouraged [67]. In the context of medical plastic waste management, Kumar et al. (2023) proposed a distinctive 5R strategy (Table 1): refuse, reduce, reuse, repurpose, and recycle. The repurpose principle encourages finding innovative uses for PET items, such as using PET bottles or containers for different applications [68].

### 3.4. The 6R Framework

The 6R framework, encompassing recover, reuse, reduce, recycle, redesign, and remanufacture technologies at different stages, efficiently diminishes waste generation from PET bottles, contributing to closed-loop models with multiple product lifecycle systems [69]. Remanufacturing PET-based products or components, such as bottles or containers, to restore them to their original quality can help retain the value of the materials and reduce the necessity for new production.

### 3.5. The 7R Framework

Shaili et al. (2021) emphasizes the pressing need for nations to embrace an integrated approach in addressing the challenges posed by plastic waste. This approach involves adopting the 7R model of sustainability (replace, redesign, re-modify, recover, repurpose, recycle, and refuse), which advocates for a comprehensive and effective disposal mechanism through various methods and processes within the CE framework [70]. An alternative 7R concept (rethink, refuse, reduce, reuse, repair, re-gift, and recycle) was proposed for plastic products used in ophthalmology, as mentioned by Gheorghe et al. [71]. Encouraging individuals to rethink their consumption choices; refuse single-use or non-recyclable products; reduce overall consumption; and adopt practices like reusing, repairing, re-gifting, and recycling can actively contribute to minimizing its environmental impact [71]. At the same time, Osman et al. outlines a different plastic waste minimization 7R strategy—recovering, repairing, reusing, reducing, re-gifting, refusing, and rethinking—to prevent the release of waste materials, including microplastics, into the environment [72].

### 3.6. The 8R Framework

Vlajic et al. proposed the 8R concept to manage food packaging waste in the agri-business sector [73]. By including principles like rethink, redesign, reduce, replace, reuse, repurpose, recycle, and recover, agri-businesses are actively advancing circularity through the adoption of recycled PET (rPET). This shift has prompted packaging producers to offer products with up to 100% rPET content [73].

### 3.7. The 9R and 10R Frameworks

Recently, the 9R and 10R frameworks aimed to optimize social, material, and economic values, with a particular emphasis on the environmental aspect (Table 1). In order to minimize plastic waste, Kassab et al. are considering an extension of the 6R framework (reduce, reuse, recycle, repair, refuse, and rethink) to a comprehensive 9R framework, incorporating refurbish, remanufacture, re-purpose, and recover strategies. For instance, the remanufacturing of ocean-retrieved PET waste or post-consumer PET bottles has been explored using various technologies, including injection molding, 3D printing, and thermoforming [36].

However, a circular economy striving to extend the useful life of post-consumer plastic waste could encompass the 9R strategies: rethink, reduce, reuse, repair, refurbish, remanufacture, repurpose, recycle, and recover. Sitadewi et al. suggests that the 9R framework should also include “refusing”, a concept closely aligned with circular economy (CE) implementation, leading to the development of a 10R framework [74]. The refuse strategy primarily involves substituting fossil-based plastics with bioplastics that serve similar functions [79]. Another 10R framework applied to plastics is outlined by Calistro Friant et al. in their work (refuse, reduce, resell/reuse, repair, refurbish, remanufacture, repurpose, recycling, recovery, and re-mine). Thus, it has introduced a novel concept of “re-mine”, which involves retrieving the plastic waste through landfill mining [75].

Among the outlined strategies, circulating materials flows by recycling has the potential to transform human-created systems, aiming for an optimal balance between economic prosperity and environmental well-being [80]. Consequently, the creation of new materials from discarded ones, such as producing screen-printed electrodes using recycled PET (polyethylene terephthalate) soft-drink bottles [81], and the regeneration of natural systems through efficient waste management, contribute to enhancing economic circularity.

## 4. PET Recycling Approaches

PET recycling stands out as a highly impactful strategy to reduce PET waste significantly and actively contributes to the smooth flow of materials within the CE. Therefore, this method goes beyond simply reducing the volume of waste, playing a crucial role in conserving energy and resources and reducing emissions [59]. PET recycling techniques were categorized into four main groups: primary, secondary, tertiary, and quaternary recycling, as outlined by various studies [25,82,83,84,85,86]. In addition, Nikles et al. (2005), Elamri et al. (2017), and Sheel et al. (2019) mention the concept of the “zero-order” technique as a potential consideration for PET recycling [44,85,86]. However, this term is not widely acknowledged in the field of PET recycling. It suggests more of a reuse approach, involving the cleaning of PET products for further use in their original form, rather than a traditional recycling process. Moreover, a growing interest in a novel approach to PET recycling in recent years is known as biological recycling, utilizing specialized enzymes [87].

**Figure 8:**
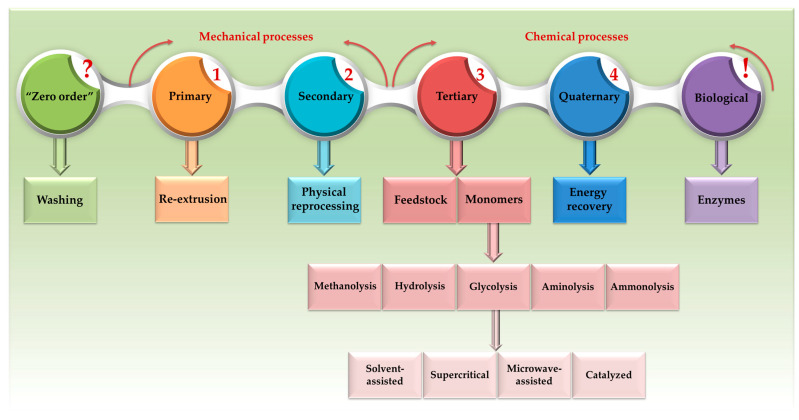
Different methods for PET recycling based on mechanical or chemical processes.

As a result, Figure 8 depicts the PET recycling methods (including the concept of “zero order”), which are briefly discussed further. It can be noted that both primary and secondary recycling rely on mechanical procedures, whereas tertiary and quaternary recycling are both based on chemical processes. Xin et al. (2021) categorize biological recycling as a type of chemical recycling [88]. Given that biological recycling encompasses enzymatic reactions and entails a fundamental chemical transformation of PET, it can be considered properly classified as a chemical process (Figure 8). By contrast, the “zero-order” method was considered a mechanical recycling only because the material is reused without altering its chemical structure (similar to primary and secondary recycling).

### 4.1. “Zero-Order” Recycling

The “zero-order” concept involves a direct recycling–reuse process for PET bottles through cleaning–washing–refilling, mirroring the approach commonly used for cleaning glass bottles. Initially, the gathered bottles undergo a rigorous hot washing process using detergents. Subsequently, specialized sensors are employed to inspect the bottles, identifying and removing those that contain volatile or liquid contaminants [86]. This approach involves reusing plastic items in their original form (e.g., reusing a mineral water bottle) [44]. Although this practice is commonly used in some countries, a significant limitation of this technique should be considered. This implies the inability to completely remove contaminants that have been absorbed by the PET material, as the plastic bottles are more prone to absorbing contaminants compared to glass [89].

### 4.2. Primary Recycling

The most traditional recycling approach, also referred to as re-extrusion/closed-loop recycling, involves the “in-plant” recycling of pristine industrial scrap materials that are free from contaminants [44]. This technology relies on mechanical reprocessing to create a product with equivalent qualities [90]. Thus, the uncontaminated scrap undergoes a specific treatment process. First, it is shredded into flakes, which facilitates its further processing. The recycled flakes can be combined with fresh, unused plastic material to produce a blend suitable for manufacturing. Alternatively, these shredded flakes can be segregated as a second-grade product. They are suitable for other molding applications that might not require the highest grade of material but still benefit from its quality [25]. The primary recycling is straightforward and cost-effective [91], making it ideal for handling a singular type of uncontaminated scrap. However, this method is not widely favored, due to the need for uncontaminated scrap and the drawback of minimizing the number of cycles for each material [55,92].

### 4.3. Secondary Recycling

In comparison to primary recycling, this approach relies on the mechanical (physical/open loop) reprocessing of contaminated plastic scraps into products with lower quality than the initial ones [84,90,91]. In physical reprocessing, PET material undergoes sorting, separation, grinding, melt filtration, and reshaping without altering the basic polymer structure [85,89]. Although the process is simple, inexpensive, and environmentally friendly [55], it presents some drawbacks, including the deterioration of the product’s characteristics (such as its molecular weight, mechanical properties, melt viscosity, and impact resistance) with each cycle [25]. Additionally, this type of recycling is considered to be unsuitable for manufacturing items intended for contact with food [85].

### 4.4. Tertiary Recycling

In tertiary recycling (also known as chemical recycling), the polymer undergoes de-polymerization by breaking down into its chemical constituents. As pointed out by Hopewell in 2009, this process leads to the recovery of valuable materials like feedstock and monomers (also illustrated in Figure 8) [90]. However, the utilization of PET for refinery feedstock has been found to result in significant amounts of corrosive benzoic acid (up to 500 g/kg PET), presenting a notable drawback, as highlighted by Meys et al. [80]. Consequently, Schwarz (2021) underscored the significance of monomer production, illustrating that tertiary recycling plays a crucial role in extracting essential materials and showcasing the potential for resource recovery within a circular economy for PET (closed loop) [84]. However, it should be mentioned that the monomers obtained after PET recycling can also be used to produce high-value products, a process known as the chemical upcycling of PET (open loop) [80,93].

**Figure 9:**
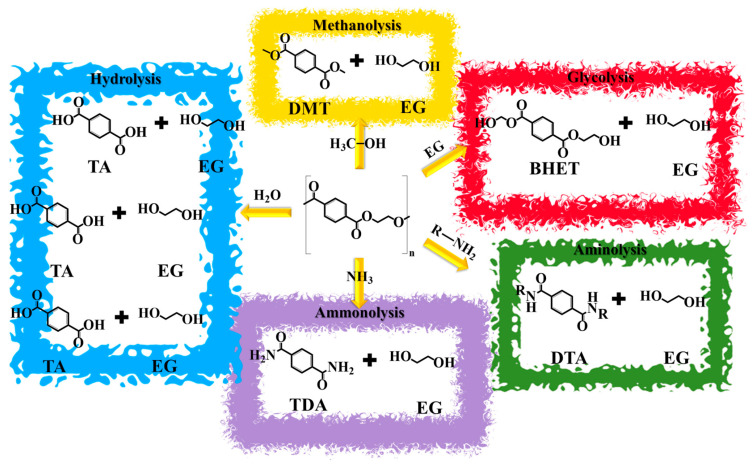
PET chemical recycling processes (methanolysis, hydrolysis, glycolysis, alcoholysis, aminolysis, and ammonolysis) and their corresponding chemical reactions.

This recycling process aims to derive monomer units by completely or partially breaking down large PET polymer chains into oligomers or monomers. These include terephthalic acid (TA), dimethyl terephthalate (DMT), bis(2-hydroxyethyl) terephthalate (BHET), and ethylene glycol (EG) [94]. The standard chemical recycling procedure involves initial treatment protocols such as PET waste sorting, cleaning, and grinding, closely resembling processes employed in mechanical recycling. Subsequently, the resulting PET pellets are introduced into a reactor to initiate the chemical depolymerization reaction [88]. Thus, chemical recycling relies on five fundamental reactions: methanolysis, hydrolysis, glycolysis, alcoholysis, and aminolysis, as identified by Paszun et al. as early as 1997 [95]. Figure 9 depicts each of the chemical reactions underlying PET depolymerization, highlighting the corresponding monomers obtained and their chemical formulas.

#### 4.4.1. Methanolysis

The methanolysis of PET involves a transesterification reaction with methanol, usually occurring under elevated temperatures (>180 °C) and high-pressure conditions (20–40 bars) [96]. This process can use liquid, superheated vapor, or supercritical methanol to produce dimethyl terephthalate (DMT) and ethylene glycol (EG) [97], as shown in Figure 9. The reaction can occur either in the absence or in the presence of catalysts, including metal acetates, metal oxides, and biomass-derived catalysts [98]. Various industries, such as Hoechst, DuPont, Dow Chemicals, and Eastman, have employed this recycling method for many years [99]. However, a significant drawback of this effective procedure lies in the corrosive nature of methanol, which can lead to a reduced lifespan of industrial installations [96,100]. Additionally, the process incurs high costs, particularly the purification of DMT and EG [97].

#### 4.4.2. Hydrolysis

As evidenced in Figure 9, the hydrolysis reaction is employed to break down the PET polymer chains in order to obtain TA and EG. This process can occur under different conditions, including acidic, alkaline, or neutral conditions, which are excellently detailed by Shojaei et al. [101]. In brief, acid hydrolysis employs strong acids, like H_2_SO_4_, H_3_PO_4_, and HNO_3_. The reaction can occur at lower temperatures and/or pressure. However, the reaction mixture is corrosive and generates a substantial volume of liquid waste containing inorganic salts [97]. Also, the extensive use of acids increases the overall cost of the process and adversely affects the purity of EG [102].

In typical alkaline hydrolysis, PET waste reacts with alkali-water solutions (usually using NaOH), under pressure for several hours. This process involves high pressure and elevated temperatures, resulting in the formation of an alkali metal salt of TA (sodium salt of TA), which is subsequently precipitated through acidification [103]. The depolymerization of PET under basic conditions is also influenced by non-aqueous alkaline hydrolysis, which employs ether solvents such as dioxane and THF in conjunction with an alcohol [99]. Guo et al. proposed an alternative innovative approach for PET waste using an alkaline hydrolysis method with reduced solvent–solid state reaction (LSR). This process transforms various PET plastic wastes into sodium terephthalate (Na_2_TP) and ethylene glycol (EG) [104]. However, the hydrolysis process faces drawbacks, such as a high operational pressure, an elevated temperature (>200 °C), and extended reaction times (3–5 h or more) required for complete PET digestion [89]. Also, the elevated cost of TA purification poses a significant barrier, limiting its use in certain industries for food-grade recycled PET production [94].

Neutral hydrolysis, regarded as environmentally friendly, involves the use of water or steam, along with water-soluble salts as catalysts [105]. Nevertheless, this type of process has its disadvantages, including potentially slower reaction rates, the need for elevated temperatures and pressures for efficient hydrolysis, and reduced effectiveness in depolymerization compared to acidic or alkaline hydrolysis, resulting in lower yields of valuable monomers. Moreover, the presence of mechanical impurities within the polymer continues to affect the purity of TA [102].

#### 4.4.3. Aminolysis

Aminolysis involves depolymerizing PET using amine aqueous solutions and diverse catalysts (e.g., zinc acetate, lead acetate, glacial acetic acid, and potassium sulfate) within a temperature range from 20 to 100 °C [101]. As evidenced in Figure 9, ethylene glycol (EG) and the corresponding diamides of terephthalic acid (DTA) are typically obtained [106]. However, an excess of ethanolamine, along with various chemicals acting as catalysts (glacial acetic acid, sodium acetate, and potassium sulfate), led to high yields of pure bis(2-hydroxy ethylene) terephthalamide (BHET) from PET sourced from waste fibers and soft-drink bottles [107]. Hoang et al. also demonstrated that excess ethylenediamine can lead to the formation of trimer bis(2-aminoethyl) terephthalamide and other higher-molecular-weight oligomers, like α,ω-aminoligo(ethylene terephthalamide) [108]. Triethylamine has been noted for achieving the highest yields of monomers (TA and EG), when compared to other amines (dimethylamine and methylamine), which exhibit lower product yields, often accompanied by intermediate products [94,109]. Despite its potential, PET aminolysis remains less explored compared to other chemical processes and is not widely implemented on a commercial scale [107].

#### 4.4.4. Ammonolysis

During PET depolymerization through ammonolysis processes, anhydrous ammonia (NH_3_) attacks PET in an ethylene glycol medium. This method is relatively slower compared to aminolysis, necessitating both a catalyst and elevated reaction pressures to enhance satisfactory reaction rates [103]. Thus, the reaction is typically conducted at temperatures ranging from 70 °C to 180 °C under 20 bar pressure, utilizing a zinc acetate catalyst [110]. This reaction aims to produce ethylene glycol (EG) and terephthalamide (TDA) as reaction products (Figure 9). TDA serves as a transitional component in the synthesis of terephthalonitrile, which can further undergo hydrogenation to yield p-Xylylenediamine or 1,4-bis(amino-methyl)cyclohexane [103]. Xie et al. effectively investigated the terephthalonitrile production from PET waste through in situ catalytic pyrolysis, employing urea as an active ammonia source and γ-Al_2_O_3_ as catalyst [111]. Despite its effectiveness, ammonolysis has garnered less attention compared to other chemical recycling approaches, as highlighted in the scientific literature [101,106].

#### 4.4.5. Glycolysis

Among the previously mentioned chemical depolymerization methods for PET, glycolysis stands out as a highly effective and promising recycling approach. Table 2 provides a comprehensive comparison of the final products, main advantages, and drawbacks associated with each chemical process employed in PET depolymerization. In addition to the primary monomers highlighted in Figure 9, Table 2 also identifies secondary products (byproducts) that can form during these chemical reactions. Thus, one may see that glycolysis offers some key benefits, including high monomer yields and purity, short reaction time (20–60 min), milder operating conditions, low volatility of ethylene glycol (EG), and minimal by-product generation [112]. These attributes make glycolysis well-suited for large-scale applications. Moreover, the use, recovery, and reusability of catalysts enhance the sustainability and resource efficiency of the glycolysis process, further supporting its viability for industrial PET recycling.

As a result, glycolysis is widely adopted by major companies, like DuPont, Dow Chemicals, Goodyear, Shell Polyester, and others, among industrially utilized PET recycling techniques [103]. The glycolysis process remains a subject of significant attention, particularly in the quest for more efficient catalysts and exploring the potential applications of the resulting compounds [82]. Due to its promising potential, the glycolysis-based PET recycling technique is thoroughly investigated in this review.

In brief, by comparing the various chemical recycling methods for PET—such as glycolysis, methanolysis, hydrolysis, aminolysis, and ammonolysis—one may observe distinct differences in conversion efficiency, environmental impact, and economic feasibility within the circular economy framework. Glycolysis stands out for its high conversion efficiency, producing ethylene glycol with minimal by-products and lower energy requirements compared to other methods. Methanolysis, while effective in yielding dimethyl terephthalate and ethylene glycol, faces challenges due to methanol’s corrosive nature and high purification costs. Hydrolysis methods vary: acidic hydrolysis offers faster reactions but generates corrosive waste, alkaline hydrolysis requires high temperatures and pressures, and neutral hydrolysis is environmentally friendly but has slower reaction rates and lower yields. Aminolysis and ammonolysis methods utilize amine and ammonia solutions, respectively, with varying degrees of efficiency and complexity, impacting their commercial viability. Overall, while each method has unique advantages and drawbacks, glycolysis appears promising due to its efficiency and suitability for large-scale implementation in the circular economy

### 4.5. Quaternary Recycling

The primary recognition of the quaternary recycling method lies in its emphasis on energy recovery [84,91]. This process is based on incineration (direct approach) or on pyrolysis (indirect approach). The incineration (combustion) is used for generating heat energy, while the pyrolysis serves the dual purpose of producing aliphatic and aromatic hydrocarbons. These hydrocarbons serve either as an alternative to fossil fuels or as a source of chemical substances [113]. Quaternary recycling is usually applied when gathering, sorting, and separating PET waste proves challenging or economically unfeasibility, or poses toxicity concerns [87]. However, since pyrolysis completely destroys the PET material, it cannot yield recyclable plastics like the other recycling methods [47].

### 4.6. Biological Recycling

In response to the increasing demand for a global circular economy, PET biological recycling has gained prominence in recent years. PET is recognized for its limited biodegradability, attributed to the aromatic terephthalate units in its molecular structure. However, it was proved that the hydrolysable ester linkages in PET (especially for amorphous PET) are susceptible for depolymerization using certain enzymes. This phenomenon led to a promising biotechnological method for recovering terephthalic acid and ethylene glycol [114]. Excellent overviews of the enzymatic degradation of PET (key enzymes and specific reaction conditions) were reported so far [87,115,116,117,118]. Briefly, PET depolymerization through the action of hydrolytic enzymes can occur either in vitro or in microbial environments [27]. Most studied enzymes for PET degradation are from the esterases class (Enzyme Commission number EC 3.1.1.), such as lipases, cutinase [119], and carboxylesterase, and PETase from PET hydrolase class (EC 3.1.1.101) discovered in 2016 [120]. Currently, the only reported industrialized enzyme for PET biological recycling is a thermostable variant metagenome-derived LC-Cutinase (LCC^ICCG^) [121]. Ding et al. (2023) suggested two approaches for rational redesign of LCC^ICCG^ enzyme by using a machine learning tool to address problematic commercial PET plastic with high crystallinity [122]. Moreover, Garcia et al. (2022) highlights that Carbios’ PET enzymatic recycling technique (C-ZYME^®^) can degrade 97% of PET in 16 h, already enabling successful manufacturing of the world’s first food-grade PET plastic bottles [87,123]. Even with the encouraging outcomes, the enzymatic recycling method is still in the early stages of development and has a significant distance to cover before reaching scale-up production, because of the generation of some undesired waste [88,124].

## 5. Glycolysis—A Way for Circular Horizons

In pursuit of a circular economy, chemical recycling is extensively promoted as a means to mitigate fossil resource depletion and reduce greenhouse gas emissions [80]. Decomposing waste into monomers serves as a fundamental element for producing fresh PET (closing the loop in the material’s life cycle). Additionally, it enables the generation of high-value new products (open-loop upcycling). Among the previously discussed tertiary recycling methods (also depicted in Figure 8 and Figure 9), glycolysis emerges as one of the most promising techniques for depolymerizing PET on an industrial scale [45]. Glycolysis offers a significant enhancement in closed-loop and weighted circularity, allowing for the production of virgin PET to be replaced directly with pristine recycled PET [46,47,125]. Hence, an examination of PET bottles’ recycling, spanning from 2020 to 2049, demonstrates that the implementation of chemical recycling via glycolysis, coupled with enhanced collection systems facilitated by recycling centers, will markedly enhance the circularity of PET bottles [126].

As shown in Figure 9, glycolysis entails incorporating ethylene glycol (EG) into PET chains, resulting in the production of bis(2-hydroxyethyl)terephthalate (BHET), along with dimers and oligomers. This usually occurs at temperatures between 180 and 240 °C and in the presence of a catalyst [127]. However, this method may also employ other glycols, like diethylene glycol (DEG) and propylene glycol (PG). In the case of using PG, the resulting products can include bis(2-hydroxypropyl) terephthalate (BHPT), BHET, and hydroxypropyl–hydroxyethyl terephthalate [128], as evidenced in Table 2.

Glycolysis offers several advantages, including simplicity, operation at atmospheric pressure and relatively low temperatures, and low volatility and non-toxicity of glycolysis reagents and products (especially the monomer BHET). In addition, it avoids the generation of acid or alkali wastewater and allows for ease of separation and purification (by hot-water extraction, cooling crystallization, and adsorption) [88]. This process yields high quantities of BHET that can be directly used in the synthesis of new recycled PET (rPET). However, the kinetics of PET glycolysis reveal that the process is exceedingly slow without a catalyst, and achieving complete depolymerization to BHET is not feasible. In addition to the BHET monomer, the final mixture contains secondary products, specifically oligomers, making the recovery of BHET challenging [128]. Furthermore, glycolysis may not be suitable for low-quality PET waste and could be limited in the recovery of valuable monomers [103]. Thus, the glycolysis process was investigated by different methods and was classified accordingly in solvent-assisted glycolysis, supercritical glycolysis, microwave-assisted glycolysis, and catalyzed glycolysis [128], as previously represented in Figure 8.

### 5.1. Solvent-Assisted Glycolysis

In glycolysis assisted by solvents, ethylene glycol (EG) facilitates the breakdown of PET within a solvent serving as a reaction medium. Notably, a successful PET glycolysis involved the addition of xylene to the EG and Zn(OAc)_2_ mixture, resulting in an 80% BHET [129]. Liu et al. explored various solvents, including aniline, nitrobenzene, NMP, and dimethyl sulfoxide (DMSO), introducing a dissolution–degradation approach that reduced the PET glycolysis reaction time to 1 min. Particularly noteworthy was the use of DMSO and zinc acetate as a catalyst [130]. However, the extensive use of organic solvents poses environmental challenges, thus hindering further research [128]. Addressing this concern, Le et al. proposed the use of anisole as an eco-friendly co-solvent alternative. Thus, it was demonstrated that anisole effectively facilitates PET conversion to BHET at a lower reaction temperature of approximately 153 °C compared to catalytic glycolysis without a co-solvent (~200 °C) [131].

### 5.2. Supercritical Glycolysis

Supercritical glycolysis involves the breakdown of PET using EG at temperatures and pressures exceeding the critical point of the polymer. While previous research has explored experimental conditions for PET methanolysis and hydrolysis, recent studies have extended these conditions to glycolysis [127,132,133,134,135]. One benefit of using supercritical fluids in these kinds of processes is that they do not require catalysts, which can be challenging to remove from the end-products. Nevertheless, there are also some disadvantages of this technology, such as the high temperature and pressure needed to complete the process [135].

### 5.3. Microwave-Assisted Glycolysis

For the depolymerization of PET via glycolysis, microwave irradiation offers several benefits over conventional techniques. It was found that microwave heating led to a decrease in activation energy, a rise in the rate reaction constant value, a consistent reduction in reaction time, and precise temperature control [136]. For example, Sangalang et al. determined the activation energy for PET glycolysis to be 29 kJ/mol, using generalized kinetics, which aligns more closely with theoretical values for ester bond degradation and transesterification. Notably, these values are significantly lower than those reported in other glycolysis kinetic studies (>80 kJ/mol) [137]. The depolymerization reaction was examined by Achilias et al. through microwave irradiation at various power levels and time intervals, leading to a 100% depolymerization rate at 150 W for two minutes, 100 W for five minutes, and only 50 W for ten minutes [138]. In another study, Chen et al. explored the influence of temperature and microwave irradiation on the kinetics of PET degradation. They achieved an activation energy of 36.5 kJ/mol, resulting in an approximately 80% BHET yield in just 35 min, employing a 500W microwave power at 196 °C [139]. To enhance the PET glycolysis process, simplified and faster approaches were investigated by combining microwave-assisted PET glycolysis with ionic liquids (IL) [136] or eco-friendly calcium oxide (CaO) [140].

### 5.4. Catalyzed Glycolysis

Despite the advantages offered by the glycolysis process, obstacles such as extended reaction times and low BHET yields hinder its widespread implementation [88]. Consequently, there is considerable interest in developing and utilizing highly active catalysts, dedicated to the enhanced depolymerization of PET. By employing a catalyst, glycolysis of PET can yield valuable intermediate BHET, which can be further recycled as a precursor for PET through repolymerization or upcycled for the synthesis of other biodegradable polymers [141].

#### 5.4.1. Reaction Mechanism

**Figure 10:**
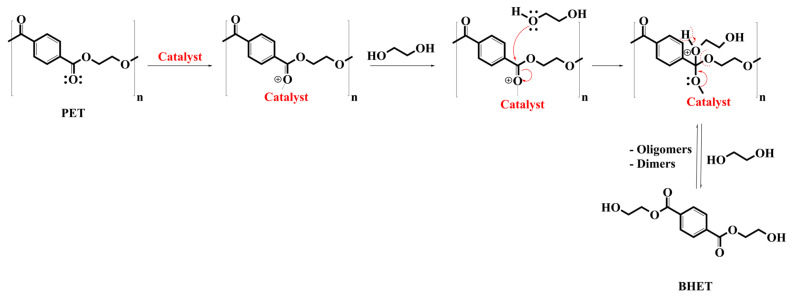
Possible glycolysis mechanism (adapted after [142,143]).

The glycolysis reaction mechanism using a catalyst is depicted in Figure 10. In brief, the reaction is initiated by the metal cations present in the catalysts, which engage with the oxygen in the carbonyl group. This interaction results in an increased partial positive charge on the carbon atom within the carbonyl group. Following this, a free electron pair on the oxygen of ethylene glycol (EG) targets the carbonyl carbon of the polyester. Subsequently, the newly formed bond between the carbonyl carbon of the polyester and the hydroxyethyl group of ethylene glycol breaks the long polymeric chain into short oligomer chains. Ultimately, this results in the formation of the BHET monomer. The interaction dynamics between the metal cation and the oxygen atom in the carbonyl group are significantly influenced by several factors. For example, Imran et al. noted that complex spinels exhibit superior catalytic performance in comparison to single metal oxides. This is attributed to their larger surface areas and higher concentrations of active sites on the catalyst surface. Moreover, the achieved catalytic yield (92%), by using ZnMn_2_O_4_ as a catalyst under certain conditions, is significantly impacted by the nature of metal cations, their coordination within the crystal structure (either tetrahedral or octahedral), and the geometry of the spinel (either tetragonal or cubic) [127]. The glycolysis reaction rate is influenced by factors such as the type and quantity of catalyst, temperature, pressure, reactant ratios, and others. Additionally, the conversion of dimer to BHET monomer is a reversible process. Upon reaching equilibrium, the depolymerization reaction tends to shift backward, increasing the amount of dimer at the expense of the BHET monomer [142,143]. Therefore, understanding the ideal parameters for the glycolysis reaction is crucial. For example, EG can easily attack PET and form BHET when metal-based catalysts are used, because the metal forms a complex with the carbonyl group.

#### 5.4.2. Types and Roles of Catalysts

**Figure 11:**
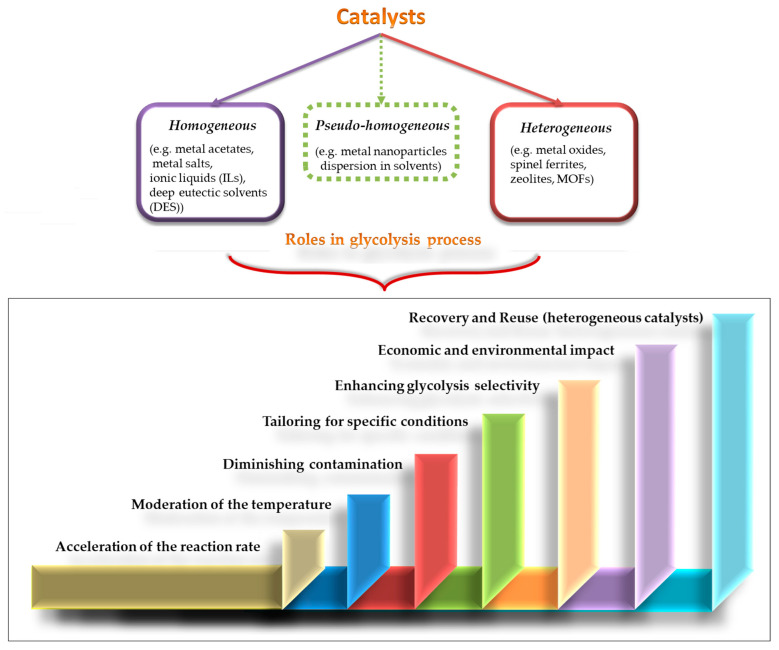
Classification of catalysts and their role in PET glycolysis process.

Recent research has extensively explored various catalysts to enhance the catalyzed glycolysis of PET, aiming to fulfill multiple key roles in the process, as illustrated in Figure 11. The primary goal of the catalyst in PET glycolysis is to accelerate the reaction rate, ensuring a more efficient process [25]. Additionally, catalysts can enable glycolytic reactions at lower temperatures (for improved energy efficiency and process control). They enhance selectivity for achieving higher yields of desired monomers, while minimizing undesired by-products. Furthermore, catalysts help minimize contamination in glycolysis products for high-quality recycled PET production. Designing or selecting catalysts tailored to specific reaction conditions allows for customization according to glycolysis process requirements [88,128,144]. In the context of the circular economy, incorporating catalysts into the PET glycolysis process enhances depolymerization efficiency (high monomer yields), cost-effectiveness, and sustainability. This is achieved through lowered overall costs, promoting environmental sustainability via PET recycling (or upcycling), enabling straightforward monomer recovery from reaction mixtures, and facilitating catalyst reuse (particularly for heterogeneous catalysts), as evidenced in Figure 11.

Furthermore, concerning the impact of catalyst types (Figure 11), the most common catalysts explored for enhancing PET glycolysis are the homogenous catalysts (e.g., metal acetates, ionic solvents, metal salts, and deep eutectic solvents), as well as heterogeneous catalysts, like metal oxides, tailored silica gels, metal–organic frameworks (MOFs), catalysts derived from biomass waste, zeolites, and others [44,45,88,145,146]. Moreover, another category of catalysts can be considered, referred to as pseudo-homogeneous catalysts, which involve solid nanoparticle dispersion in solvents. For example, the effectiveness of a cost-efficient pseudo-homogeneous nanocatalyst, graphite carbon nitride, in PET glycolysis was demonstrated by Wang et al. [147]. Contrasting with the homogeneous catalysis, heterogeneous catalytic systems, which are known for their advantages, like ease of separation, non-toxicity, and stability [144], among others, have been extensively investigated. Therefore, this review aims to emphasize the benefits of heterogeneous catalysis compared to homogeneous catalysis, with further focus on a specific class of heterogeneous catalysts, namely oxide-based catalysts.

#### 5.4.3. Heterogenous vs. Homogenous Catalysis

Most of the homogenous catalysts (encompassing metal acetates [146,147,148,149,150], chlorides [151,152], alkoxides [46], hydroxides [153], carbonates [154,155], sulfates [156], and phosphates [157]) necessitate an additional unit operation in the chemical process, such as distillation. Zinc acetate (Zn(OAc)_2_) has been widely employed as a catalyst for the glycolysis of PET, demonstrating its effectiveness for polyesters degradation (82 documents found on Scopus when we searched for “PET AND glycolysis AND zinc AND acetate”, March 2024). Since 1989, zinc acetate has been successfully utilized as a catalyst in the glycol depolymerization of PET, and the resulting BHET product was purified and re-polymerized to generate new polyesters [49,158]. Other metal acetate-based catalysts, such as Mn(OAc)_2_, Co(OAc)_2_, and Pb(OAc)_2_, were investigated but proved to have lower catalytic activity than Zn(OAc)_2_ [148,159]. Yet, a recent study has demonstrated that the catalytic efficiency of tropine surpasses that of zinc acetate at 170 °C and remains comparable at 190 °C [160].

As highlighted in Table 3, while homogeneous catalysts proved to have impressive catalytic efficiency, they come with drawbacks, like difficulties in catalyst separation/reusability and limited selectivity and purity of the end product [50,127]. Conversely, heterogeneous catalysis is considered to be more effective and aligns with the principles of sustainable chemistry, and eco-friendly and improved catalytic processes [145]. Thus, using a heterogeneous catalyst can offer several advantages, such as high stability, non-corrosivity, and effective removal. These factors are essential for reducing contamination in resulting monomers and achieving high yields of pure monomer [161,162,163,164]. In addition, it should be noted that the advantage of heterogeneous catalysts to be reused in further glycolysis processes (unlike homogeneous ones) makes them economically favorable [144].

Thus, various types of heterogeneous catalysts have been investigated recently for enhancing PET glycolysis, from metal oxides with different basicity [162,166], zeolites [167], spinel ferrites [50], metal–organic frameworks (MOFs) [168], multiwalled carbon nanotubes (MWCNT) [169], and biomass waste-derived heterogeneous catalysts [170]. However, oxide-based compounds stand out as economically viable catalysts due to their robust mechanical strength and straightforward preparation, making them particularly well-suited for large-scale applications [50].

## 6. Oxide-Based Catalysts

Oxides can directly participate as active catalysts in the PET glycolysis process, but they can also serve as a support structure for other catalytically active species. The selection of particular oxides relies on their characteristics, responsiveness, and appropriateness for the intended heterogeneous catalytic processes. Various operational factors, such as temperature, catalyst quantity, mixing approach, alcohol/oil molar ratio, feedstock purity, and reaction duration, play a fundamental role in this selection [164].

### 6.1. Oxides as Active Catalysts

#### 6.1.1. Pure and Mixed Metal Oxides

Metal oxides could be a preferable option for glycolysis catalysts over traditional ones. Their advantages include an elevated monomer yield, robust mechanical strength, high melting points, adaptability for use in fixed and fluidized beds, a regenerability potential, ease of separation, and prolonged durability [127]. As represented in Table 4, different pure (e.g., Fe_2_O_3_, Nb_2_O_5_, ZnO, Fe_3_O_4_, and CoO) and mixed (e.g., ZnO–Fe_3_O_4_, CeO_2_-Fe_3_O_4_, ZIF-8-Fe_3_O_4_, and Co/RZnO) metal oxides were investigated as active catalysts for enhancing PET depolymerization by glycolysis. However, the utilization of superparamagnetic γ-Fe_2_O_3_ nanoparticles resulted in a BHET monomer yield higher than 90% but required an elevated temperature of 300 °C (Table 4) [171]. Nabid et al. demonstrated an improved BHET yield (100%), using a bifunctional catalyst based on γ-Fe_2_O_3_ and N-doped graphene [172] at 195 °C. Recently, in the presence of organic ligands immobilized on mesoporous silica (SiO_2_), Fe_2_O_3_ metal-oxide nanoparticles exhibited a superior performance compared to bare Fe_2_O_3_ nanoparticles, Fe^3+^ ion, and homogeneous FeCl_3_ salts (with equivalent Fe loading) at 190 °C, but with a slightly lower BHET yield (Tabel 4) [173]. Son S.G. et al. showcased the enhanced performance of another metal oxide, namely MnO_2_ ultrathin exfoliated nanosheets (e-MON), achieving a BHET yield of 100% in a 30-minute reaction at 200 °C, surpassing the efficiency of bare bulk MnO_2_ (77.6%). Additionally, they demonstrated its outstanding reusability across five cycles [174].

Combining two oxides increases the number of catalytic sites by modifying the electronic structure of active metals, thereby enhancing the interaction between the substrate and catalyst and consequently accelerating the reaction rate [146]. As observed in Table 4, mixing metal oxides has been proved to present different behavior regarding catalytic activity, as compared with pure metal oxides. For instance, Yun et al. (2023) explored PET depolymerization using ZnO and Fe_3_O_4_. However, the utilization of a composite oxide derived from them, specifically ZnO–Fe_3_O_4_, in the form of magnetic hollow micro-sized nanoaggregates (HMNAs), revealed a synergistic effect. This resulted in a significantly ultrahigh monomer yield of 92.3% within a brief period of 30 min at 190 °C.

This outcome surpassed the individual performances of ZnO and Fe_3_O_4_ nanoparticles [175]. In the same study, various mixed oxides-based hollow micro-sized nanoaggregates (HMNAs), including CeO_2_–Fe_3_O_4_ and ZIF-8-Fe_3_O_4_, were examined, revealing improved catalytic properties and recyclability [175]. Similar behavior was observed by Fuentes et al. when they investigated recovered zinc oxide (RZnO) and cobalt oxide (RCoO) from spent alkaline and Li-ion batteries. The mixed oxide (Co/RZnO) led to a higher BHET monomer yield (80%), as compared to 50% and 10% for RZnO and RCoO, respectively. This was explained by the large number of weak acid sites and the formation of strong acid sites, as well as a synergetic effect between Co_3_O_4_ and ZnO [176]. Cao et al. showed that Co/ZnO and Mo/ZnO ultrathin nanosheets exhibited enhanced catalytic activity compared to pure ZnO at 180 °C after only 60 min [177]. These studies showcased the superior performance of these mixed oxide systems in glycolysis, indicating their potential for efficient catalysis and ease of recovery for subsequent applications. Also, the substitution of Fe for Al led to significantly higher PET conversion rates, highlighting the potential of Mg-Fe oxides as a biocompatible catalyst for PET chemical recycling, yielding non-toxic BHET suitable for various applications, including food and beverage packaging [162].

#### 6.1.2. Spinel Ferrites

Ferrites, which belong to the spinel materials category, typically exhibit magnetic characteristics, facilitating their magnetic separation [179]. Despite their well-known catalytic qualities, they have not been thoroughly studied for the catalytic glycolysis of PET. However, CoFe_2_O_4_, NiFe_2_O_4_, CuFe_2_O_4_, and ZnFe_2_O_4_ synthesized via an environmentally friendly, solvent-free, and straightforward mechanochemical method, were investigated as catalysts for PET glycolysis. Their catalytic performance was correlated with the M^2+^ ions’ Lewis acid strength (in the order ZnFe_2_O_4_ > CuFe_2_O_4_ > CoFe_2_O_4_ > NiFe_2_O_4_). Thus, ZnFe_2_O_4_ demonstrated the highest activity in PET depolymerization to produce BHET, with a monomer yield of 79% and complete PET conversion at 195 °C, after 150 min [50]. Moreover, Wang et al. employed ionic liquid surfactants to modify CoFe_2_O_4_ nanoparticles, resulting in complete PET conversion and an approximately 96% BHET monomer yield at 195 °C (150 min). These catalysts offer the benefit of easy removal via an external magnet and can be reused for up to 10 cycles without diminishing their catalytic efficacy [180].

Magnetite (Fe_3_O_4_) is a significant member of the spinel ferrite family and has been explored as a catalyst for converting PET into BHET monomer. Jo et al. demonstrated that Fe_3_O_4_ obtained through coprecipitation displayed superior glycolysis performance compared to the decomposition or hydrothermal methods. It achieved a PET conversion close to 100% and a BHET yield of 93.5% at 195 °C for 2 h. This finding suggests that Fe_3_O_4_ catalytic activity is effective for producing recycled PET (r-PET) through PET waste glycolysis [181].

#### 6.1.3. Zeolites

Zeolites are crystalline aluminosilicates with microporous structures, featuring cavities of 0.3-1.5 nm [182], which exhibit catalytic activity due to their unique structure and composition. Thus, the presence of metal ions or acidic sites in zeolite frameworks has the potential to improve PET glycolysis efficiency [106]. Two zeolites, SiO_2_/AlO_2_, with a ratio of 1:5 (β-zeolite) and 4:5 (Y-zeolite), respectively, were employed as transesterification catalysts for PET bottle wastes and presented good catalytic activities for BHET monomer yield (~65%) after 7 h [167]. In another study, Lee et al. demonstrated that two-dimensional zeolite nanosheets are capable of effectively depolymerizing PET at relatively low temperatures, achieving a PET conversion of over 60% at 140 °C and over 98% at 180 °C after 1 h. However, the yield of bis(hydroxyethyl) terephthalate (BHET) surpassed only 50%. The reaction batches contained various types of polyols, including monomeric BHETs and oligomeric BHETs, which were directly utilized in the production of remanufactured polyurethane foam (PUF) [183]. The findings enable an environmentally friendly PET waste depolymerization process into pure BHET, suitable for synthesizing valuable chemicals.

#### 6.1.4. Polyoxometalates (POMs)

Two studies have highlighted the employment of polyoxometalates (POMs) as catalysts in PET glycolysis. POMs represent a diverse category of metal oxides with different shapes and sizes, demonstrating a remarkable range of physicochemical properties [184]. Geng et al. investigated different transition metal-substituted POMs, such as K_6_SiW_11_MO_39_(H_2_O), where M represents Zn^2+^, Mn^2+^, Co^2+^, Cu^2+^, or Ni^2+^, as a catalytic system for PET depolymerization under mild conditions [185]. Among these, K_6_SiW_11_NiO_39_(H_2_O) presented the highest catalytic activity, leading to complete PET degradation and a BHET yield of 84% in just 30 min. Additionally, K_6_SiW_11_MnO_39_(H_2_O) demonstrated significant involvement in PET degradation (~78%) [185]. In another study, Fang et al. examined a sandwich-structure form of transition metal-substituted POMs (Na_12_ [WZnM_2_(H_2_O)_2_(ZnW_9_O_34_)_2_] (M = Zn^2+^, Mn^2+^, Co^2+^, Cu^2+^, and Ni^2+^). These POMs, featuring multiple transition-metal active sites, exhibited an outstanding catalytic performance in PET glycolysis under mild conditions [186]. These studies highlight promising catalysts for PET degradation, characterized by mild conditions, rapid reaction kinetics, low energy consumption, and high stability. In accordance, these features make them a viable option for industrial-scale PET recycling.

#### 6.1.5. MOFs

Metal–organic frameworks (MOFs), categorized as coordination polymers, typically consist of a metal-oxide core bonded with organic linkers. These have gained attention for their potential applications in heterogeneous catalysis [187]. Therefore, three MOF catalysts, namely ZIF-8, ZIF-67, and MOF-5, were synthesized and applied in the glycolysis of poly(ethylene terephthalate) (PET). All catalysts exhibited notable catalytic performances. Among them, ZIF-8 demonstrated the highest efficacy, achieving complete PET degradation and a BHET yield of approximately 77% at 197 °C after 90 min [188].

Wang et al. improved the performance of catalytic activity of MOFs by loading magnetic nanoparticles (CoFe_2_O_4_) using a bimetallic zeolitic imidazolate framework (ZIF-8/ZIF-67) [189]. The bimetallic MOFs proved to have a more unique performance compared to single metal-based ZIF-8 in the PET glycolysis. Moreover, the CoFe_2_O_4_@ZIF-8/ZIF-67 composite exhibited an improved catalytic performance in the glycolysis of PET in comparison to ZIF-8/ZIF-67, leading to an 88.5% BHET monomer yield in 60 min (195 °C). It should be mentioned that this type of composite catalyst was evaluated for the first time as a catalyst for the degradation of PET/PBT mixed plastic, leading to successful conversion into monomers [189].

Recently, Yun et al. showed that two-dimensional (2D) MOFs nanosheets obtained by using high-gravity RPB (ZIF-L-RPB), presented excellent heterogeneous catalytic activity for the glycolysis of poly(ethylene terephthalate) (PET) compared to nanosheets prepared in a stirred tank reactor (ZIF-L-STR) [190]. As a result, PET was degraded with a conversion rate of 99.4%, and the yielded BHET was 93.9% within a 30-min period, utilizing only a minimal amount of 0.2 wt.% at 195 °C [190]. These findings suggest that MOFs can offer encouraging potential for being used as cost-effective heterogeneous catalytic applications.

### 6.2. Oxides as Catalytic Support

In addition to their role as active catalysts, oxide-based compounds can serve as supporting structures for other catalysts in PET glycolysis reactions. For instance, γ-Al_2_O_3_ alumina has been utilized to support various metal oxides with differing levels of basicity, including calcium, cerium, or cobalt oxides. Among these catalysts, 10%Ce/Al_2_O_3_ demonstrated complete conversion of PET waste with superior selectivity toward the main product BHET, while maintaining high catalytic efficiency even after five consecutive runs [166]. Another example is provided by Zhang et al., who utilized porous MgAl_2_O_4_ spinel material as a support for Mn_3_O_4_ metallic oxide. Notably, the highest BHET yield of 97.6% was achieved at 190 °C for 3 h using the Mn_3_O_4_/p-spMgAl800 catalyst, with an EG/PET ratio of 20. Remarkably, the catalytic activity remained consistent even after five consecutive recycling runs and persisted following the third periodic regeneration sequence [191].

## 7. Conclusions

PET thermoplastic is widely utilized in packaging due to its advantageous properties, such as transparency, food safety, durability, and cost-effectiveness. However, the significant increase in PET production has led to adverse environmental impacts, including pollution and resource depletion, with projections indicating further escalation. To mitigate these issues, various scenarios, ranging from 3R to 10R (which encompass reduce, reuse, recycle, recover, repurpose, etc.), were envisioned lately for effective management of PET waste within a circular economy framework. These scenarios aim to minimize the environmental impact, reduce landfill waste, and conserve energy and raw materials.

PET recycling stands as a highly impactful strategy for significantly reducing waste and promoting material flow within the circular economy. Various PET recycling approaches, including glycolysis and primary; secondary, tertiary, and quaternary recycling, alongside innovative techniques like “zero-order” and biological recycling, were evaluated for sustainable waste management. Chemical recycling, especially glycolysis, is heavily promoted to mitigate fossil resource depletion and greenhouse gas emissions. Glycolysis, which is promising for depolymerizing PET on an industrial scale, enhances closed-loop circularity by replacing virgin PET with recycled PET.

Given this context, a substantial interest in developing new catalysts to enhance the PET depolymerization rate and selectivity in BHET production by glycolysis was observed. These catalysts proved to have the capability to produce significant amounts of BHET, a valuable intermediate for r-PET production or the synthesis of other biodegradable polymers. Notable catalytic activity has been observed in pure and mixed metal oxides, spinel ferrites, zeolites, polyoxometalates, and MOFs. Additionally, oxide-based compounds serve also as supporting structures for other catalytic active phases in PET glycolysis reactions, ensuring the complete conversion of PET waste with high selectivity toward the main product, BHET. Consequently, recycling PET materials to create circular flows has the potential to balance economic prosperity with environmental well-being.

## Figures and Tables

**Table 1:** Different R strategies proposed for plastic (especially PET) management in the context of CE.

R-Imperatives	Strategies	References
3R	Reduce, reuse, and recycle	[59,61]
4R	3Rs and recover	[62,63,64,65,66]
5R	3Rs and redesign and recover	[62]
	3Rs and refuse and rot	[67]
	3Rs and refuse and repurpose	[68]
6R	4Rs and redesign and remanufacture	[69]
7R	Replace, redesign, re-modify, recover, repurpose, recycle, and refuse	[70]
	3Rs and rethink, refuse, repair, and re-gift	[71]
	Recover, repair, reuse, reduce, re-gift, refuse, and rethink.	[72]
8R	4Rs and rethink, redesign, replace, repurpose,	[73]
9R	4Rs and rethink, repair, refurbish, remanufacture, and repurpose	[36]
10R	9Rs and refuse	[74]
	4Rs and refuse, repair, refurbish, remanufacture, repurpose, and re-mine	[75]

**Table 2:** Comparison of the chemical recycling processes, highlighting the main advantages and disadvantages [94,95,96,97,98,99,100,101,102,103,104,105,106,107,108,109,110].

Chemical Process	Advantages	Disadvantages	Primary Products (Monomers)	Secondary Products (Byproducts)
Methanolysis	- Efficient recovery of EG and methanol - High final product quality - Easy purification steps	- Severe reaction conditions - High costs of monomer purification - Corrosive nature of methanol	DMT, EG, alcohols, phthalate derivatives	TPA, derivative alcohols, BHET, unsaturated polyester resins, epoxy resins and its hardeners, vinyl ester resins, alkyd resins
Acidic hydrolysis	- Recovery of EG - Lack of side reactions - Short time reaction	- Use of strong acids - High operational costs - Resulting inorganic salts and aqueous wastes - Dependent on PET particle size/shape - Requires distillation process to separate acid from EG	TPA, EG, oxalic acid (from HNO_3_ reagent)	BHET, PET, unsaturated polyester resins, epoxy resins, vinyl ester resins, and alkyd resins
Neutral hydrolysis	- Environmentally friendly - Absence of organic solvents	- TPA needs extra purification - High operational costs related to high temperatures and pressure - Unsuitable for industrial scale	Contaminated TPA and EG	BHET, PET, unsaturated polyester resins, epoxy resins, vinyl ester resins, and alkyd resins.
Alkaline hydrolysis	- Suitable for PET waste with high content of impurities - Cost-effective by comparison to the acid and neutral hydrolysis	- High pressure and elevated temperatures - Difficulties in TPA and catalysts separation process	TPA, EG, salts	BHET, PET, unsaturated polyester resins, epoxy resins, vinyl ester resins, and alkyd resins
Aminolysis	- Mild reaction conditions (T < 100 °C) - High yield and purity of the products - Use of catalysts for higher monomer yields	- Not used for industrial applications - Requires further reactions to by-product removal	EG, mono- and di-amines of TPA	Unsaturated polyester resins, epoxy resins, non-ionic polymeric surfactants
Ammonolysis	- Depolymerization at low pressure - Using catalysts (Zn(OAc)_2_) for higher yields	- Long reaction time - High temperatures (120–180 °C) - Ammonia is corrosive and toxic	EG and TPA-diamine	Terephthalonitrile, p-Xylylenediamine, and other derivatives
Glycolysis	- Short reaction time (20–60 min) - High monomer yields - Monomers of high purity - Milder operating conditions - Cost-effectiveness - Catalyst recovery and reusability - Large-scale applications	- High temperatures - Requires several filtration steps for monomer recovery	BHET, BHET dimer and oligomers, BHPT, hydroxypropyl–hydroxyethyl terephthalate, oligoester diols	PET, oligomers

**Table 3:** Differences between homogenous and heterogeneous catalysis processes [50,127,164,165].

No.	Process Characteristics	Homogeneous Catalysis	Heterogeneous Catalysis
1	Reaction time	Fast reaction time	Moderate reaction time
2	Monomer conversion	High	Moderate
3	Water presence influence	Sensitive	Not sensitive
4	Catalyst distribution	Same phase with reactants	In different solid phase
5	Catalyst recovery	Difficult to recover (usually neutralized, leading to waste chemical production)	Easy separation
6	Catalyst reuse	Not possible	Reusable
7	Catalyst recycling	Difficult	Possible
8	Purification	Extensive purification steps	Easy purification
9	Costs	Expensive	Potentially cheaper
10	Durability	Short life	Long life

**Table 4:** Pure and mixed metal oxides used as active catalysts for PET glycolysis.

Oxide-Based Catalyst	PET Conversion ^1^	BHET Yield ^2^ (%)	Optimum Conditions		Ref.
	$\left(\frac{W_{0}–W_{1}}{W_{0}}\times 100\%\right)$	$\left(\frac{W_{BHET}/M_{BHET}}{W_{0}/M_{PET}}\times 100\%\right)$	Temperature (°C)	Time (min)	
γ-Fe_2_O_3_		>90	300	60	[171]
γ-Fe_2_O_3_ γ-Fe_2_O_3_/N-doped graphene	100	~40	195	180	[172]
	100	100	195 (250)	180 (80)	
Fe_2_O_3_ (ligand–silica supported)	99	70	190	-	[173]
Nb_2_O_5_	100	85	195	220	[48]
MnO_2_ e-MON (exfoliated MnO_2_ nanosheets)	97.8	88.4	200	60	[174]
	100	100		30	
ZnO	-	79.2	180	40	[175]
Fe_3_O_4_	-	<15	180	40	
ZnO–Fe_3_O_4_	100	92.3	190	30	
CeO_2_-Fe_3_O_4_	100	95.4	197	45	
ZIF-8-Fe_3_O_4_	100	85.2	190	20	
RZnO	-	50	196	120	[176]
RCoO		10			
Co/RZnO		80			
ZnO	55	51.7	180	60	[177]
Co/ZnO	100	93.2			
Mo/ZnO	100	94.5			
Mg-Fe-l.s.c.	97.4 ± 2.6	68.7 ± 11.0	200	60	[162]
Mg-Fe-h.s.c.	96.8 ± 3.2	68.7 ± 11.0			
Mg-Al-l.s.c.	98.8 ± 0.1	84.0 ± 0.0			
Mg-Al-h.s.c.	64.0 ± 10.7	52.1 ± 9.3			

^1^ *W*_0_ and *W*_1_ are the initial and un-depolymerized mass of PET; ^2^ *W_BHET_* represents the mass of BHET, while *M_BHET_* the *M_PET_* are the molecular weights for BHET and PET’ repeating unit (254 g/mol and 192 g/mol, respectively) [178].

## Data Availability

Not applicable.
